# Cancer survival in Cixian of China, 2003–2013: a population‐based study

**DOI:** 10.1002/cam4.1416

**Published:** 2018-03-13

**Authors:** Dongfang Li, Daojuan Li, Guohui Song, Di Liang, Chao Chen, Yachen Zhang, Zhaoyu Gao, Yutong He

**Affiliations:** ^1^ Cixian Cancer Institute Handan 056500 China; ^2^ Cancer Institute The Fourth Hospital of Hebei Medical University/The Tumor Hospital of Hebei Province Shijiazhuang 050011 China

**Keywords:** Cixian, population‐based cancer registry, relative survival, screening, upper gastrointestinal cancer

## Abstract

Cixian is one of the high‐risk areas for upper gastrointestinal cancer in China and the world. From 2005, comprehensive population‐based screening for upper gastrointestinal cancers has been conducted in Cixian. The aim of this study was to investigate population‐based cancer survival from 2003 to 2013 and to explore the effect of screening on upper gastrointestinal cancer survival in Cixian. Observed survival was estimated using the life table method. The expected survival from the general population was calculated using all‐cause mortality data from the population of Cixian with the EdererII method. Cixian cancer registry, with a total coverage of 6.88 million person years, recorded 19,628 cancer patients diagnosed during 2003–2013. In Cixian, from 2003 to 2013, there were 19,628 newly cancer cases and 13,984 cancer deaths, with an incidence rate of 285.37/100,000 and mortality rate of 203.31/100,000. The overall five‐year relative cancer survival for patients diagnosed in Cixian in 2003–2013 was 22.53%. The relative survival for all cancers combined in Cixian had an overall upward trend from 2003 to 2013. Among upper gastrointestinal cancer in Cixian, the five‐year relative survival for cardia gastric cancer was highest at 30.42%, followed by oesophageal cancer at 25.37% and noncardia gastric cancer at 18.93%. In 2013, the five‐year relative survival for oesophageal cancer, cardia gastric cancer, and noncardia gastric cancer patients aged 45–69 years was 39.97% (95% CI: 34.52–45.43%), 51.74% (95% CI: 42.09–60.86%), and 37.43% (95% CI: 26.93–48.17%), respectively, the absolute values increasing 14.11%, 16.71%, and 14.92% compared with that in 2003. There is an increasing trend in overall survival for upper gastrointestinal cancer with early screening and treatment of cancer in Cixian.

## Introduction

Cixian is one of the high‐risk areas for upper gastrointestinal cancer in China and the world. The Cixian Cancer Registry was the first population‐based cancer registry in Hebei Province and was established in 1974. Studies on cancer incidence and mortality trends in Cixian have previously been reported in detail 1, 2, 3, 4, 5. Data for cancer incidence and mortality in Cixian were published in Cancer Incidence in Five Continents (CI5VIII, CI5X and CI5XI) 6, 7, 8. From 2005, comprehensive screening for upper gastrointestinal cancer, based on the population aged 45–69, was conducted throughout the whole county. Remarkably, the incidence of oesophageal cancer in Cixian has shown a sharp downward trend with the support of the Chinese government and extensive efforts for cancer prevention and control 5. Reliable information on cancer survival in the general population is critical to measuring the overall effectiveness of cancer diagnosis and management, including access to effective treatments, which can then be used by healthcare planners and professionals. However, there is currently little cancer survival data from Cixian. This study was designed to examine upper gastrointestinal cancer survival profile and its relationship to cancer screening in Cixian. Standardized quality control and analytical methods to describe cancer survival and analyze the change in upper gastrointestinal cancer survival after the cancer screening program were used.

## Materials and Methods

### Data sources

Hebei Provincial Cancer Registry Centre is responsible for compiling cancer data collections, evaluations, and publications from local population‐based cancer registries. The population‐based Cixian Cancer Registry was established in 1974 and covered a population of 6,878,097 among the 11 years. Cancer information was reported to cancer registries by local hospitals and community health centers, including the Basic Medical Insurances for Urban Residents and the New‐Rural Cooperative Medical System.

Data for patients diagnosed with cancer were obtained from the Cixian Cancer Registry. Individuals with the first occurrence of primary cancer and multiple primary cancers were selected. There were 19,628 new cancer patients diagnosed during 2003–2013. Cancer records were excluded if they were based only on a death certificate or unknown vital status. After exclusions, 18,786 patients were included in the survival analysis (95.7% of those eligible). The rate of follow‐up was 97.2%. Data on the population used to calculate relative survival were obtained from the Cixian Bureau of Statistics.

A mix of active and passive follow‐up methods was used to identify the vital status and cause of death for all registered patients from the date of diagnosis until 31 December 2016. Passive reporting relies on the periodical linkage of cancer registration records with death records from any cause from the mortality surveillance system. Active reporting involves registry personnel examining the sources of data and abstracting the required information on vital status on special forms from different types of hospitals and health insurance systems. Home visits or telephone contacts with next of kin were used for patients whose vital status could not be ascertained by other methods.

The national screening for upper gastrointestinal cancer program has been available in Cixian since 2000. From 2005, the first round of comprehensive population‐based screening for upper gastrointestinal cancer was conducted in Cixian. In the past decade, more than 50,000 high‐risk residents aged 45–69 years among the whole county were screened with endoscopy. The positive rate is between 2.2% and 3.0%, and the early diagnostic rate is between 85.0% and 92.0%. Moreover, the treatment rate is very high with 90.0%. The screening for upper gastrointestinal cancer can improve the survival for high‐risk patients.

According to “Guideline of Chinese Cancer Registration,” we checked the data quality using the inclusion criteria from “Cancer Incidence in Five Continents Volume X,” which was required by the International Agency for Cancer Registry (IACR) and International Agency for Research on Cancer (IARC). The quality and completeness of the cancer registry data were assessed with IARC‐crgTools to identify errors, inconsistencies, and unusual combinations of cancer site, morphology, sex, and age at diagnosis 9. All information on primary cancer site and histology was coded according to the International Classification of Diseases for Oncology, 3rd edition (ICD‐O‐3) and the International Statistical Classification of Diseases and Health Related Problems 10th Revision (ICD‐10). Cases of oesophageal cancer (ICD10: C15) and stomach cancer (ICD10: C16) were extracted and analyzed from the overall cancer database. Stomach cancer was assigned by subsite: cardiac cancer (ICD10: C16.0) and noncardia gastric cancer (ICD10: C16.1–C16.9).

### Statistical methods

Relative survival is the ratio of the observed proportion surviving in a group of patients to the expected proportion that would have survived in a comparable group of people from the general population. Observed survival was estimated using the life table method 10. The expected survival from the general population was calculated using all‐cause mortality data from the Cixian population with the EdererII method 11, which is also a life table method. Individual cases were stratified by period, gender, age group, and cancer site. One‐year, three‐year, and five‐year relative survival was estimated. Abridged life tables were smoothed to complete (single‐year‐of‐age) life tables and extended to the age of 99 using the Elandt‐Johnson method 12. Relative survival during 2003–2010 was used by the cohort method. Period analysis was used to calculated relative survival for the period 2011–2013, because five‐year relative survival for patients diagnosed during 2011–2013 were not available for all patients (Fig. S1). All analyses were performed using SAS version 9.2 (SAS Institute, Cary NC) and Stata version 12.0 (StataCorp LP, College Station TX).

## Results

### Overall relative cancer survival in Cixian

In Cixian, from 2003 to 2013, there were 19,628 newly cancer cases and 13,984 cancer deaths, with an incidence rate of 285.37/100,000 and mortality rate of 203.31/100,000. During this decade, incidence and mortality fluctuated over time.

By statistical analysis, the overall one‐year, three‐year, and five‐year relative cancer survival for patients diagnosed in Cixian in 2003–2013 was 63.59% [95% confidence interval (CI) 62.87–64.31%], 33.84% (95% CI: 33.12–34.57%), and 22.53% (95% CI: 21.87–23.19%), respectively. For males, the overall five‐year relative survival for all cancers combined [20.31% (95% CI: 19.48–21.16%)] was 20.70% lower than that for females [25.61% (95% CI: 24.56–26.66%)]. According to age at diagnosis, the overall five‐year relative survival for cancer patients younger than 45 years, 45–69 years, and older than 70 years was 28.80% (95% CI: 26.53–31.10%), 24.63% (95% CI: 23.82–25.45%), and 14.59% (95% CI: 13.35–15.88%), respectively. The relative cancer survival for patients younger than 45 years was the highest at all times. The overall relative survival for all cancers combined declined with age. (Table 1).

**Table 1:** Incidence and mortality rates and relative survival (RS, %) for all cancers combined in Cixian, 2003–2013

Year	Cases	Incidence Rate (1/100,000)	Deaths	Mortality Rate (1/100,000)	One‐year RS (95% CI)	Three‐year RS (95% CI)	Five‐year RS (95% CI)
2003	1614	267.82	1138	188.84	62.58 (60.02–65.04)	28.89 (26.51–31.32)	21.39 (19.18–23.69)
2004	1696	280.79	1221	202.15	65.09 (62.57–67.51)	34.83 (32.3–37.39)	28.32 (25.84–30.86)
2005	1785	293.53	1257	206.71	57.46 (54.94–59.90)	31.18 (28.78–33.62)	26.54 (24.18–28.99)
2006	1799	292.53	1360	221.14	58.77 (56.24–61.22)	33.95 (31.48–36.45)	30.11 (27.64–32.64)
2007	1886	302.20	1369	219.36	62.45 (60.06–64.76)	38.66 (36.24–41.10)	34.43 (32.01–36.88)
2008	1839	292.20	1300	206.56	61.98 (59.56–64.32)	35.33 (32.94–37.74)	30.52 (28.17–32.93)
2009	1866	294.17	1302	205.25	62.05 (59.65–64.36)	35.15 (32.81–37.52)	30.70 (28.38–33.07)
2010	1787	280.76	1147	180.21	62.81 (60.40–65.13)	31.43 (29.14–33.76)	28.09 (25.81–30.42)
2011	1818	284.36	1297	202.87	64.42 (62.04–66.70)	32.77 (30.48–35.09)	28.77 (26.52–31.07)
2012	1774	276.76	1311	204.53	67.32 (65.01–69.53)	35.18 (32.74–37.64)	30.39 (28.01–32.82)
2013	1764	274.02	1282	199.15	70.54 (68.23–72.73)	41.50 (38.91–44.09)	37.19 (34.54–39.86)
Total	19,628	285.37	13,984	203.31	63.59 (62.87–64.31)	33.84 (33.12–34.57)	22.53 (21.87–23.19)

The overall five‐year relative cancer survival for patients diagnosed in Cixian was 26.62% (95% CI: 25.42–27.84%), 30.95% (95% CI: 29.77–32.15%), and 32.05% (95% CI: 30.64–33.47%) in different period: 2003–2006, 2007–2010, and 2011–2013, respectively. There is an obvious increase trend.

### Relative survival by cancer types

**Figure 1:**
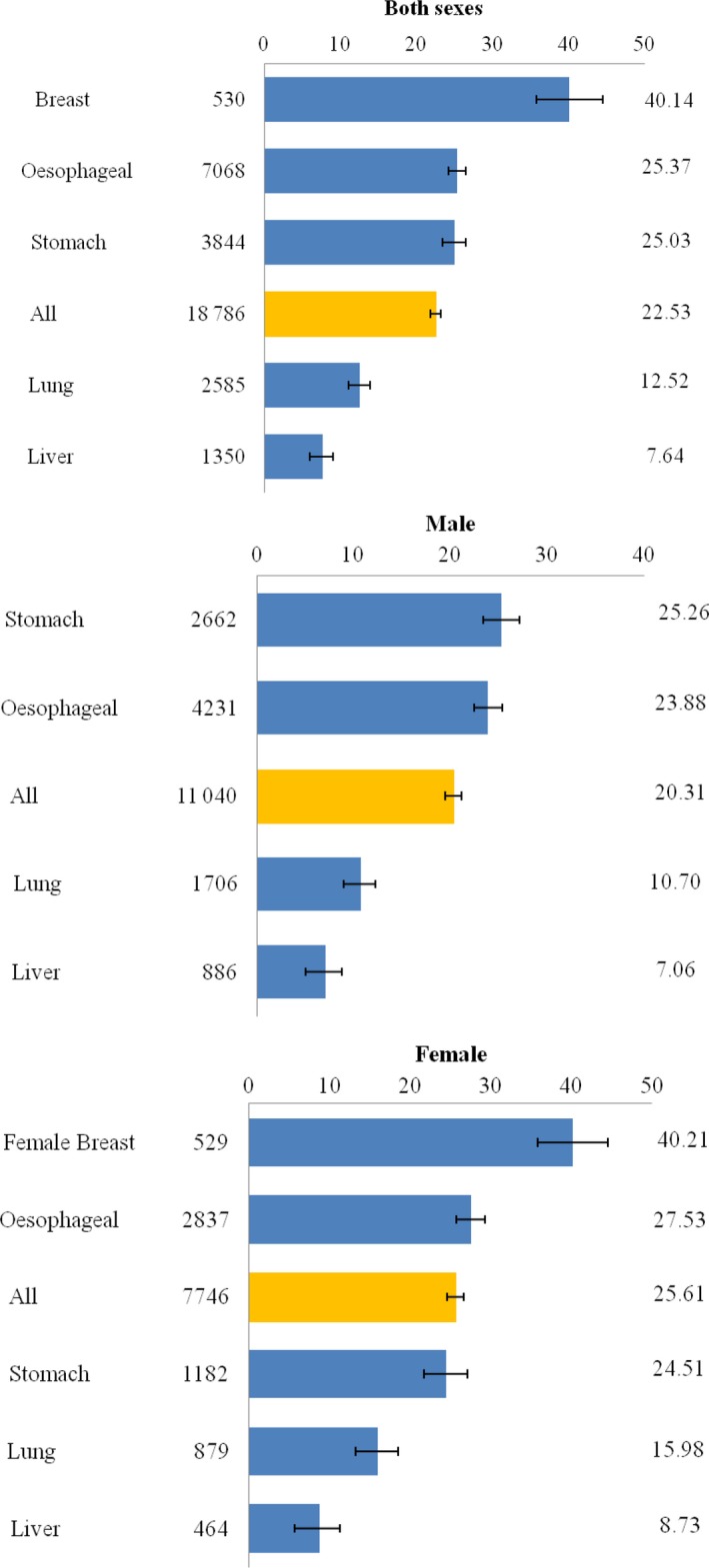
Five‐year relative survival (RS, %) by cancer type in Cixian, 2003–2013.

The five‐year relative survivals for the five most common cancers (oesophageal, stomach, lung, liver, and colorectal) in Cixian were lower than 30% in both sexes. Female breast cancer had the highest survival (40.21%), followed by cancers of the colorectum (26.07%), esophagus (25.37%), and stomach (25.03%), with lung and liver cancer, respectively, having the poorest survival (12.52% and 7.64%). In males, the five‐year relative survival for the five most common cancers ranged from 7.06% (95% CI: 5.37–9.06%) for liver cancer up to 25.26% (95% CI: 23.42–27.14%) for stomach cancer. In females, the five‐year relative survival for female breast cancer was the highest at 40.21% (95% CI: 35.85–44.55%), which was higher than 30%. The five‐year relative survival for the five other most common cancers (esophageal, stomach, lung, liver, and colorectal) ranged from 8.73% (95% CI: 6.22–11.79%) for liver cancer to up to 30.42% (95% CI: 25.60–35.42%) for colorectal cancer (Fig. 1).

The five‐year relative survival is listed for common cancers in males and in females (Fig. 1). Five‐year relative survival was highest for cancers of the ovary (42.50%) and thyroid (40.47%), and lowest for cancers of the bone (10.35%) and liver (7.64%). Cancers with relatively good prognosis (five‐year relative survival of more than 30%) included cancers of the ovary, thyroid, breast, testis, cervix, and bladder, which accounted for 5.9% of all cancers. Cancers with a poor prognosis (below 15%) were cancers of the pancreas, lung, larynx, leukemia, bone, and liver, which accounted for 24.64% of all cancer cases (Fig. 1).

### Relative survival for upper gastrointestinal cancer in Cixian

**Figure 2:**
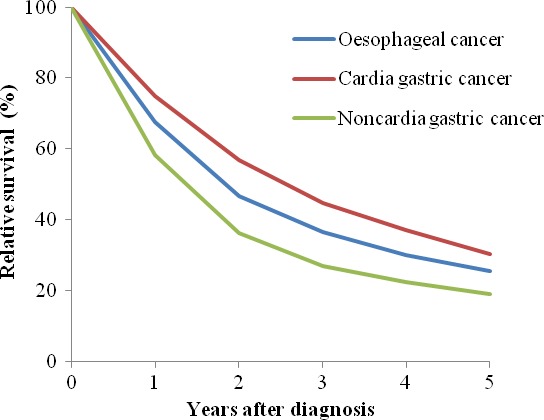
Relative survival for upper gastrointestinal cancer in Cixian, 2003–2013.

**Figure 3:**
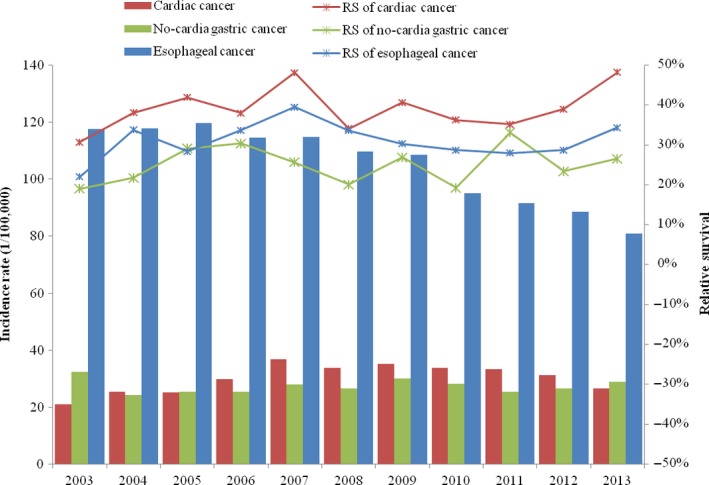
Incidence rates and five‐year relative survival for upper gastrointestinal cancer in Cixian, 2003–2013.

Upper gastrointestinal cancer is a critical public health problem in Cixian that represents 58.1% of all cancers. We further analyzed the relative survival of oesophageal cancer, cardia gastric cancer, and noncardia gastric cancer in Cixian. The five‐year relative survival for cardia gastric cancer was highest at 30.42% (95% CI: 28.20–32.69%), followed by esophageal cancer at 25.37% (95% CI: 24.24–26.51%), and noncardia gastric cancer at 18.93% (95% CI: 16.98–20.98%) (Table 2, Figs 2 and 3).

**Table 2:** Incidence cases and five‐year relative survival (RS, %) for upper gastrointestinal cancer in Cixian, 2003–2013

Year	Oesophageal cancer				Cardiac cancer				No‐cardia gastric cancer			
	All ages		45–69 years		All ages		45–69 years		All ages		45–69 years	
	Cases	RS (95% CI)	Cases	RS (95% CI)	Cases	RS (95% CI)	Cases	RS (95% CI)	Cases	RS (95% CI)	Cases	RS (95% CI)
2003	688	22.04 (18.67–25.65)	465	25.86 (21.69–30.27)	126	30.74 (22.15–40)	100	35.03 (25.13–45.38)	193	19.03 (13.28–25.72)	141	22.51 (15.58–30.38)
2004	693	33.77 (29.81–37.83)	483	36.93 (32.29–41.62)	151	38.08 (29.39–47.04)	116	40.94 (31.17–50.76)	147	21.76 (14.79–29.84)	101	22.55 (14.46–31.95)
2005	700	28.45 (24.69–32.39)	446	29.88 (25.38–34.55)	149	41.91 (32.6–51.39)	101	45.44 (34.51–56.17)	150	29.18 (21.29–37.77)	98	32.27 (22.61–42.57)
2006	673	33.72 (29.7–37.84)	454	35.46 (30.75–40.24)	178	38.06 (30.08–46.27)	128	41.34 (32.02–50.66)	149	30.36 (22.28–39.11)	88	33.29 (23.01–44.2)
2007	700	39.49 (35.42–43.59)	489	43.63 (38.85–48.37)	224	48.18 (40.59–55.63)	175	51.52 (43.14–59.5)	164	25.64 (18.76–33.19)	110	30.96 (22.11–40.38)
2008	674	33.6 (29.62–37.67)	460	35.63 (30.99–40.33)	211	34.12 (27.09–41.45)	153	38.07 (29.9–46.35)	164	20.08 (13.85–27.29)	92	27.46 (18.35–37.53)
2009	670	30.29 (26.47–34.23)	448	34.1 (29.48–38.82)	224	40.63 (33.42–47.9)	171	43.32 (35.23–51.31)	173	26.9 (19.95–34.47)	118	26.18 (18.22–34.98)
2010	607	28.78 (24.87–32.85)	417	34.21 (29.4–39.13)	212	36.26 (29.21–43.49)	164	40.13 (32.11–48.17)	165	19.28 (13.18–26.39)	105	26.16 (17.78–35.47)
2011	580	28.00 (24.42–31.72)	387	33.26 (28.69–37.93)	201	35.23 (28.41–42.26)	146	39.22 (31.37–47.12)	156	33.15 (25.19–41.50)	105	39.12 (29.01–49.30)
2012	566	28.76 (24.85–32.82)	371	36.17 (31.14–41.27)	185	38.99 (31.55–46.53)	137	44.23 (35.57–52.72)	169	23.44 (16.67–31.04)	95	28.61 (19.61–38.41)
2013	517	34.40 (29.97–38.95)	335	39.97 (34.52–45.43)	170	48.21 (39.68–56.53)	113	51.74 (42.09–60.86)	183	26.54 (19.52–34.22)	116	37.43 (26.93–48.17)
Total	7068	25.37 (24.24–26.51)	4755	28.21 (26.85–29.59)	2031	30.42 (28.20–32.69)	1504	33.78 (31.21–36.39)	1813	18.93 (16.98–20.98)	1169	22.06 (19.58–24.65)

### Relative survival for upper gastrointestinal cancer by age group in Cixian

**Figure 4:**
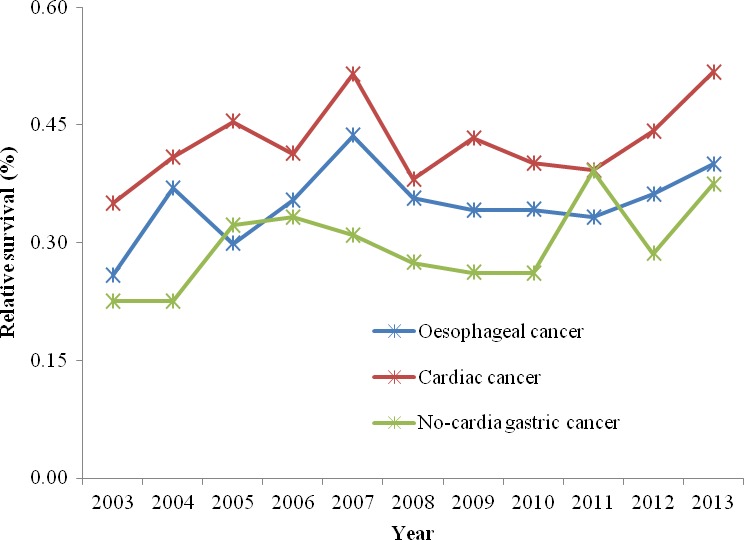
Five‐year relative survival for upper gastrointestinal cancer patients (45–69) in Cixian, 2003–2013.

Cixian, as a demonstration base for early detection and treatment of upper gastrointestinal cancer, has carried out screening for many years. Residents aged 45–69 years were screened with endoscopy. Thus, we estimated the survival for upper gastrointestinal cancer patients aged 45–69 years in Cixian. In 2013, the five‐year relative survival for oesophageal cancer, cardia gastric cancer, and noncardia gastric cancer patients aged 45–69 years was 39.97% (95% CI: 34.52–45.43%), 51.74% (95% CI: 42.09–60.86%), and 37.43% (95% CI: 26.93–48.17%), respectively, the absolute values increasing 14.11%, 16.71%, and 14.92% compared with that in 2003 (Table 2, Fig. 4).

In period, the overall five‐year relative survival for oesophageal cancer patients aged 45–69 years in Cixian in 2003–2006, 2007–2010, and 2011–2013 was 32.09% (95% CI: 29.81–34.41%), 37.08% (95% CI: 34.70–39.46%), and 36.37% (95% CI: 33.48–39.29%), respectively. For cardia gastric cancer patients aged 45–69 years, the overall five‐year relative survival was 40.74% (95% CI: 35.71–45.79%), 43.47% (95% CI: 39.36–47.56%), and 44.64% (95% CI: 39.64–49.58%) in 2003–2006, 2007–2010, and 2011–2013, respectively, while they were 26.98% (95% CI: 22.54–31.65%), 27.68% (95% CI: 23.26–32.31%), and 34.72% (95% CI: 28.89–40.69%), respectively, for noncardia gastric cancer patients aged 45–69 years in these three periods.

## Discussion

The Cixian Cancer Registry, an important component of the National Central Cancer Registry (NCCR) of China, has been collecting cancer data since 1974. In 1996, the Cixian Cancer Registry joined the International Association of Cancer Registries (IACR). Because of the accurateness of the Cixian cancer data, the incidence and mortality data were published in Cancer Incidence in Five Continents (CI5VIII, CI5X, and CI5XI). With the importance of cancer surveillance increased, data with the follow‐up information required by NCCR for surveillance of cancer survival were collected by the Cixian Cancer Registry. Survival data from Cixian were included in CONCORD‐2 13. As Cixian was found to be one of the high‐risk areas for upper gastrointestinal cancers, in both China and the world, a cancer prevention and control site was created in the 1970's. Primary prevention, secondary prevention and tertiary prevention cancer research have been systematically carried out. This study systematically analyzed cancer survival in Cixian, aiming to inform survival research and health policy priorities.

This research showed that the one‐year relative survival for all cancers combined in Cixian was 63.59% in 2003–2013. This value had significantly increased by 73.70% compared to the one‐year relative survival in 2000–2002 (36.61%) 14. The five‐year relative survival from all cancers combined in Cixian was 22.53%, which was slightly higher than the average level in rural areas of China (21.8%) 15 and higher than for Cixian in 2000–2002 (18.18%). The relative survival for all cancers combined in Cixian had an overall upward trend. This trend is closely associated with improvements in living standards and health consciousness but is lower than the five‐year relative survival of 66.4% for the United States (2006–2012) 16 and 66% for Australia (2006–2010) 17. One reason for the lower cancer survival between Cixian and developed countries relates to the different cancer spectra. Esophageal cancer, stomach cancer, lung cancer, and liver cancer were the most common cancers and the leading causes of cancer in Cixian with poor prognosis. Together, these four cancers accounted for 79% of all cancers in Cixian and were the critical reason for low cancer survival in China. Additionally, the relative survival for all cancers combined for males is lower than for females in Cixian, which is consistent with national figures.

By comparing the relative survival of common cancers between Cixian and rural Chinese areas, the five‐year relative survival for both lung cancer and liver cancer in Cixian were, respectively, 11.79% and 21.27% higher than average levels in rural areas. However, the five‐year survival for both colorectal cancer and female breast cancer in Cixian was lower than in Chinese rural areas. Early screening and treating with an enteroscope for colorectal cancer and the Two Cancers Screening Project for females must be strengthened in Cixian.

Upper gastrointestinal cancers were the most common cancers in Cixian, China. A recent study showed that the five‐year relative survival for oesophageal cancer and stomach cancer was 20.9% and 27.4%, respectively, in China for 2003–2005. The five‐year relative survival for oesophageal cancer and stomach cancer was 19.1% and 32.5%, respectively, in urban Chinese areas, and 21.2% and 24.9%, respectively, in rural Chinese areas 15. The latest U.S. data show that the five‐year relative survival for esophageal cancer and stomach cancer was 20.5% and 31.1%, respectively, in 2006–2012 16. We can see that the five‐year relative survival for esophageal cancer was 25.37% in Cixian, which was 19.67% higher than in rural Chinese areas, and was 34.95% higher than in three cities of Liaoning Province in 2000–2002 (18.8%) 18. This figure was also much higher than in Cixian in 2000–2002 (21.65%). Stomach cancer was slightly higher in Cixian (25.03%) than in rural Chinese areas, which was similar to Qidong in Jiangsu Province in 2003–2007 (26.67%) 19. The five‐year relative survival for stomach cancer in Cixian increased by 29.35% from 2000 to 2002 (19.35%) to 2003–2013 (25.03%). These findings indicated that we had achieved great success in the screening, early detection and treatment of cancer in Cixian.

Cardia gastric cancer is currently classified as gastric cancer. Lower cardia gastric cancer has been given much attention because of its increasing incidence, which is in striking contrast with the steady decrease in distal stomach cancer. In China, cardia gastric cancer has unique epidemiological features distinguishing it from distal stomach cancer. Cardia gastric cancer should be considered a distinct entity 20. In a previous study, we observed a significantly increased trend for cardia gastric cancer in Cixian with annual percentage change (APC) of 16.76% from 1988 to 1996 and 2.74% from 1996 to 2013. However, the incidence rate of noncardia gastric cancer decreased with APC of −5.25% from 1988 to 2001. It is obvious that cardia gastric cancer incidence was much higher than that for noncardia gastric cancer throughout the period 1988–2013 1. In this study, cardia gastric cancer accounted for 52.84% of gastric cancers, while the proportion of both noncardia gastric cancers and unknown specific gastric cancers was 47.16%. For cardia gastric cancer, the incidence rate was increasing and the proportion was high; however, for noncardia gastric cancer, incidence has decreased.

In this study, we analyzed the relative survival for esophageal cancer, cardia gastric cancer, and noncardia gastric cancer in Cixian. The five‐year relative survival for cardia gastric cancer is the highest among upper gastrointestinal cancers. Regarding the time trend, the survival curve for noncardia gastric cancer changed slightly, but the relative survival for cardia gastric cancer and esophageal cancer increased. Moreover, the five‐year relative survival of patients aged 45–69 years significantly increased from 2003 to 2013. Our data showed that the five‐year relative survival for esophageal cancer, cardia gastric cancer, and noncardia gastric cancer patients aged 45–69 years was 39.97%, 51.74%, and 37.43%, respectively, in 2013, all of which were higher than in 2003. Obviously, early screening and detection for cardiac cancer and oesophageal cancer were significantly effective for patients’ survival. Upper gastrointestinal cancer was accounted for 58.1% of all cancers in Cixian. With the efforts of screening for upper gastrointestinal cancer in Cixian, the survival for upper gastrointestinal cancer presented the obviously up‐trend, which had contributed substantially to the increase in overall cancer survival in Cixian.

The relative survival for cardia gastric cancer is the highest among upper gastrointestinal cancers, which is consistent with that in Linxian 21, 22. As we all know, since the 1990s, endoscopy has been widely applied in high‐risk areas of esophagus cancer, which greatly improves the diagnosis of esophagus cancer and cardiac cancer. Cixian, as one of high‐risk areas of esophagus cancer in China, carried out long‐term cancer prevention and health education and extensive screening of upper gastrointestinal cancer. Cardia biopsy by endoscopic are taken routinely, according to Guidelines for Standardized Diagnosis and Treatment of Upper Gastrointestinal Cancer, while no‐cardia gastric biopsy are taken when the lesions occur in no‐cardia gastric site. So, cardiac cancer is easier to identify than esophagus cancer and no‐cardia gastric cancer. With the support of the National Fund for Early Diagnosis and Treatment of Upper Gastrointestinal Cancer, a large‐scale endoscopic screening has been carried out on the population aged 45–69 in Cixian since 2005, but the sensitivity of endoscopy for early gastric adenocarcinoma screening is far lower than that for esophagus squamous cell carcinoma 23. Maybe this is one major reason that the survival for cardiac cancer is much higher than that for noncardia gastric cancer.

As mentioned above, Cixian has been chosen as a demonstration base for the early detection and treatment of upper gastrointestinal cancers for 17 years. Increased efforts have been invested into screening and early detection to identify precancerous lesions in upper gastrointestinal cancers, especially esophageal epithelium dysplasia, gastric intraepithelial neoplasia, preinvasive carcinoma, and gastrointestinal mucosal carcinoma. The national screening program, which uses endoscopy with mucosal iodine staining and index biopsy combined with pathological examination to confirm the disease, has been available in Cixian since 2000. From 2005 to 2014, the first round of comprehensive population‐based screening for upper gastrointestinal cancer was conducted in Cixian. Residents aged 45–69 years throughout the county were screened with endoscopy, and the second round of screening started in 2014.

Endoscopy with mucosal iodine staining was not only a sensitive technique for identifying clinically relevant upper gastrointestinal cancers but also an effective preventive technique. The sensitivity and specificity of endoscopy were approximately 80% and 93.4%, respectively 24. Wei et al. 25 that the endoscopy screening carried out in Cixian substantially reduced mortality rates. Residents aged 45–69 years were recruited from communities with high rates of oesophageal squamous cell carcinoma in Cixian. Participants in the intervention group were screened once by endoscopy with Lugol's iodine staining, and those with dysplasia or occult cancer were treated. Then, 3319 (48.62%) participants from an eligible population of 6827 were screened in the intervention group. Next, 797 volunteers from an eligible population of 6200 in the control group were interviewed. Finally, 652 incident and 542 fatal esophageal squamous cell carcinomas were identified during the 10‐year follow‐up. A reduction in cumulative mortality in the intervention group versus the control group was apparent (3.35% vs. 5.05%). The intervention group had a significantly lower cumulative incidence of esophageal squamous cell carcinoma versus the control group (4.17% vs. 5.92%). This result showed that endoscopic screening and intervention significantly reduced esophageal cancer mortality. A study by Chen et al. 26 indicated that there was a 28% reduction in the risk of gastric cancer mortality with endoscopic screening, which has important implications for gastric cancer screening in rural Chinese areas.

Upper gastrointestinal cavity, especially esophageal and cardiac cavities, could be clearly seen by the endoscope. Pathological tissue of the esophagus and cardia can also be identified for relatively more precise diagnosis by biopsy and treatment decisions. Screening with an endoscope can prevent the development of upper gastrointestinal cancers and death in high‐risk individuals, thereby contributing to life expectancy and increasing survival for upper gastrointestinal cancer, especially oesophageal cancer and cardia gastric cancer. Our study also validated that early detection and treatment of precursor lesions with an endoscope has made progress with the disease; tumor stages were changed and survival time for esophageal and cardia gastric cancers was particularly prolonged in the screened age group.

This is the first time that survival data based on the population of Cixian, Hebei Province were fully analyzed. However, there are some limitations to the study. Although the overall quality of cancer data was reliable and the long‐term survival trend can be fully shown, cancer patients in Cixian were followed until 2016. We did not obtain the five‐year survival for cancer patients diagnosed after 2011. We just analyzed the survival with period approach now. But with the development of research methods and improvements in cancer data quality, more accurate and complete survival data from Cixian will be reported.

Although there was an upward trend in cancer survival after long‐term efforts for cancer prevention and control in Cixian, the five‐year survival from all of the five most common cancers (esophageal, stomach, lung, liver, and colorectal) was below 30%. A commentary highlighted that there are substantial barriers to health care that are faced by the Chinese population, which relate to access to care, fatalism about cancer, and traditional medicine, each of which is likely to provide additional explanations for the disparities in survival between China and more developed countries 27. In a sense, this result can explain the low survival in Cixian. However, there was an obvious increasing trend for upper gastrointestinal cancer survival, especially cardia gastric cancer and esophageal cancer survival, due to the sensitivity of endoscopic technique with mucosal iodine staining in Cixian. The early detection, screening, and treatment of upper gastrointestinal cancers are significantly effective and should be carried out in the long term.

## Conflict of Interest

The authors declare no potential conflicts of interest.

## Supporting information


**Figure S1**. Survival methods used in the present study.
