# Supplementary figures and images for: Cancer survival in Cixian of China, 2003–2013: a population‐based study
Source: Cancer Med. 2018 Mar 13;7(4):1537–45. doi: 10.1002/cam4.1416 (PMC5911577; doi:10.1002/cam4.1416)

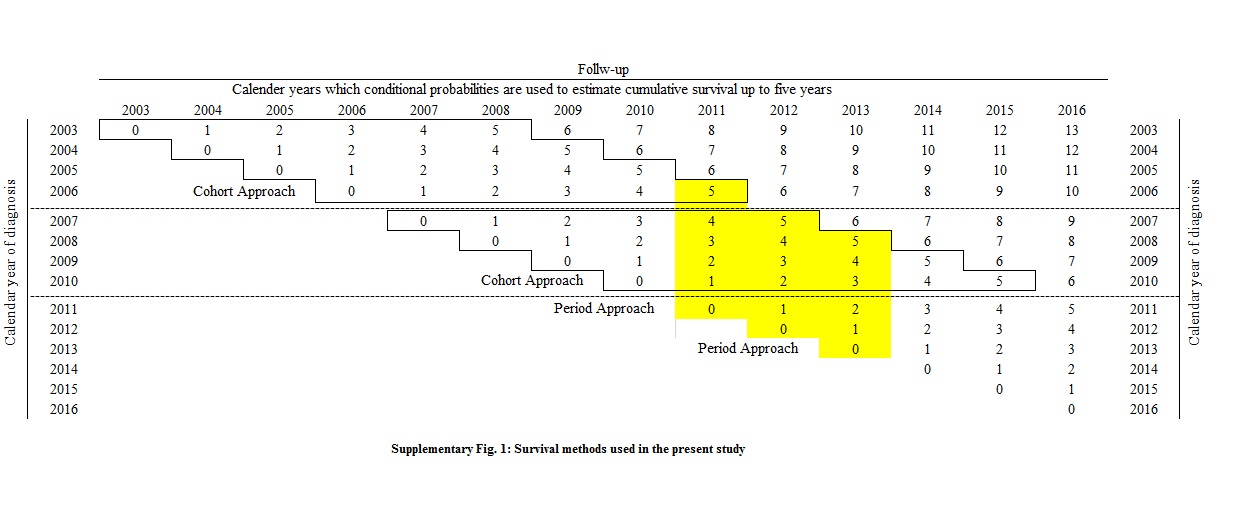

Supplement: Supplementary file 1 — Figure S1. Survival methods used in the present study. [file CAM4-7-1537-s001.jpg]
